# Diversity and Patulin Production of *Penicillium* spp. Associated with Apple Blue Mold in Serbia

**DOI:** 10.3390/jof11030175

**Published:** 2025-02-21

**Authors:** Tatjana Dudaš, Pietro Cotugno, Dragana Budakov, Mila Grahovac, Vera Stojšin, Milica Mihajlović, Antonio Ippolito, Simona Marianna Sanzani

**Affiliations:** 1 Department of Plant and Environmental Protection, Faculty of Agriculture, University of Novi Sad, Trg Dositeja Obradovića 8, 21000 Novi Sad, Serbia; dragana.budakov@polj.edu.rs (D.B.); mila.grahovac@polj.edu.rs (M.G.); vera.stojsin@polj.edu.rs (V.S.); 2 Biology Department, University of Bari Aldo Moro, Via Amendola 165/A, 70126 Bari, Italy; 3 Institute of Pesticides and Environmental Protection, Banatska 31b, Zemun, 11080 Belgrade, Serbia; milica.mihajlovic@pesting.org.rs; 4 Dipartimento di Scienze del Suolo, della Pianta e degli Alimenti, Università degli Studi di Bari Aldo Moro, Via Amendola 165/A, 70126 Bari, Italy; antonio.ippolito@uniba.it (A.I.); simonamarianna.sanzani@uniba.it (S.M.S.)

**Keywords:** apple blue mold, *Penicillium expansum*, *Penicillium crustosum*, *Penicillium solitum*, *Penicillium chrysogenum*, patulin

## Abstract

Apple blue mold, caused by the *Penicillium* species, is a significant postharvest disease, leading to food loss and impacting food safety due to mycotoxin contamination. This study aimed to identify the *Penicillium* species associated with apple blue mold in Serbia, assess their pathogenicity, and evaluate their patulin production potential. A total of 70 *Penicillium* isolates were collected from symptomatic apple fruit and identified as *P. expansum* (92.9%), *P. crustosum* (4.3%), *P. solitum* (1.4%), and *P. chrysogenum* (1.4%). The pathogenicity assay revealed *P. expansum* strains as the most virulent. Molecular detection of *msas* gene and HPLC analysis confirmed patulin production exclusively in *P. expansum* isolates. Principal Component Analysis (PCA) grouped *P. expansum* strains in two distinctive clusters, while *P. crustosum* strains clustered separately with *P. solitum* and *P. chrysogenum*, yet in distinct positions. This is the first report of *P. solitum* and *P. chrysogenum* as causal agents of apple blue mold in Serbia. The results of the study provide insights that might be useful in the development of effective control strategies for apple blue mold, ensuring consumption of healthy and safe apple fruit and apple-based products.

## 1. Introduction

Apple (*Malus domestica* Borkh.) is the largest fruit crop of temperate regions and one of the most popular fruits worldwide. Apple fruit is highly appreciated due to its nutritional value, since it is rich in vitamins, minerals, fibers, and polyphenols [1,2]. It can be stored for up to a year in a cold and controlled atmosphere, ensuring year round availability for consumption and trade. However, many postharvest pathogens can deteriorate fruit during storage, resulting in notable economic losses [3,4].

Apple blue mold is the most prevalent and economically important postharvest apple disease [5] caused by different *Penicillium* species, including *P. aurantiogriseum*, *P. brevicompactum*, *P. carneum*, *P. chrysogenum*, *P. commune*, *P. crustosum*, *P. glabrum*, *P. griseofulvum*, *P. polonicum*, and *P. solitum*, with *P. expansum* being the most common and aggressive species [6,7,8,9,10,11,12]. *Penicillium expansum* infections are not only responsible for economic losses in the apple industry, but also pose a threat to public health, due to patulin production. Patulin is a mycotoxin with mutagenic, immunotoxic, and neurotoxic activity [13]. The European Commission [14] regulated maximal patulin levels in apple-based food products: 10 μg/kg for baby food, 25 μg/kg for purees, and 50 μg/kg for juices.

In Serbia, apple is one of the most widely grown fruits and a strategic export commodity. In 2022, apples were cultivated on 27,253 ha, with a total production of 486,215 tons [15]. Previous studies indicated *P. expansum* and *P. crustosum* as causal agents of apple blue mold in Serbia [16,17]. However, the diversity of *Penicillium* spp. associated with apple blue mold is believed to be more extensive. Furthermore, to the best of our knowledge, there are no data regarding patulin production potential of *Penicillium* spp. causing apple blue mold in Serbia. Generally, research on patulin occurrence in Serbia is scarce, and there were no studies regarding the occurrence of patulin in food until 2017, when Torović et al. [18] analyzed the presence of patulin in infant fruit juice, purée, and juices. Patulin was detected in some of the analyzed samples, but under the legal limit. In the next study, patulin was detected in 51% of all examined juices, with apple juice (74% at 6.4 μg/kg) having higher contamination extent compared to multi-fruit juice (28% at 2.1 μg/kg) and 0.7% of samples with patulin above the legal limit [19].

Therefore, the aim of this study was to (i) identify the causal agents of apple blue mold in Serbia, (ii) assess the diversity of *Penicillium* spp. through morphological and genetic characterization, (iii) evaluate virulence through a pathogenicity assay, and (iv) determine the patulin production potential using molecular and HPLC analyses.

## 2. Materials and Methods

### 2.1. Sampling and Fungal Isolation

Apple fruit with blue mold symptoms were collected from 26 storage locations in Serbia, 3–6 months after the harvest, during 2020 and 2021. Fruit were surface-disinfected with 70% ethanol. Fungi were isolated from internal tissues by removing fragments, including the margin of the lesion and the healthy surrounding tissue, and placing them on potato dextrose agar (PDA and from Merck, Darmstadt, Germany) plates. After 7 days of incubation in the dark at 24 ± 2 °C, fragments from developed colonies were transferred to new PDA plates to obtain pure cultures. Microscopic morphological characteristics were observed using a KRÜSS MBL 2000 microscope (A. Krüss Optronic, Hamburg, Germany) at 40× magnification. Monosporic cultures were obtained and stored at 4 ± 1 °C on PDA slants.

### 2.2. Morphological Analysis

Growth pattern and colony morphology of the isolates were assessed as described by Visagie et al. [20] on Malt Extract Agar (MEA), Czapek Yeast Extract Agar (CYA), Yeast Extract Sucrose Agar (YES), and additionally on PDA. Three plates per medium and per isolate were seeded in three points with 10 µL of the conidial suspension (10^6^ conidia/mL). The conidial suspension of the pathogen was prepared in sterile distilled water with Tween 20 (0.05%) from 7-day-old colonies. Plates were incubated for 7 days in the dark at 25 ± 1 °C. Colony orthogonal diameters were measured (mm), and colony appearance, reverse color, and presence of exudate were recorded.

### 2.3. Molecular Identification and Phylogeny

For molecular identification of the isolates, the DNA was extracted from 7-day-old cultures grown on PDA according to a CTAB-based protocol [21]. The DNA was quantified and evaluated for its purity by a spectrophotometer (Epoch Biotek, Agilent Technologies, Santa Clara, CA, USA). Portions of the β-tubulin (*BenA*) and calmodulin (*CaM*) genes were amplified using Bt2a/Bt2b [22] and CMD5/CMD6 [23] according to Visagie et al. [20]. Single PCR reaction mix (50 µL) contained 50 ng DNA, 1X SuperHot MasterMix (Bioron, Römerberg, Germany), and 0.2 µM of each primer. PCR amplification was carried out on a Surecycler 8800 Thermal Cycler (Agilent Technologies, Santa Clara, CA, USA) as follows: initial denaturation (94 °C for 5 min), 35 cycles of denaturation (94 °C for 45 s), annealing (55 °C for 45 s), and elongation (72 °C for 1 min), followed by the final elongation (72 °C for 7 min). Electrophoresis was run on 1.5% agarose gel made with 1× SB (Sodium Boric Acid, Merck, Darmstadt, Germany) Buffer and stained with ethidium bromide (Merck, Darmstadt, Germany). The amplicons were visualized under UV light using a gel electrophoresis visualizing system (Vilber Lourmat, Eberhardzell, Germany) and sequenced (Macrogen Europe BV, Amsterdam, The Netherlands). FinchTV 1.4.0 software (Geospiza, Inc., Seattle, WA, USA) was used to evaluate the sequences’ quality, and MULTALIN software (http://multalin.toulouse.inra.fr/multalin/, accessed on 23 May 2024) was used to align the sequences. Obtained consensus sequences were compared with reference sequences available in the GenBank database using the NCBI BLAST search engine [24] and deposited in the GenBank (Supplementary Table S1).

For phylogenetic analysis, sequences were concatenated and aligned using the ClustalW algorithm [25] in MEGA X software 10.2.6 [26]. A phylogenetic tree was constructed with the maximum likelihood method using the Tamura–Nei model [27] with 1000 bootstrap replications. The phylogenetic tree was visualized using ChiPlot (https://www.chiplot.online/, accessed on 30 May 2024) [28]. Reference sequences of the *Penicillium* spp. from GenBank were included for comparison and a sequence of *Hamigera avellanea* was used as the outgroup (Supplementary Table S1).

### 2.4. Pathogenicity Assay

Apple fruit (cv. Golden Delicious) uniform in size, ripeness, and color and without physical injuries were disinfected in 2% sodium hypochlorite for 2 min, rinsed in running tap water for 1 min, and left to dry. Two wounds (3 mm × 3 mm) were made with a sterile nail on the opposite sides of each fruit in the equatorial area. Each wound was inoculated with 10 µL of conidial suspension (10^6^ conidia/mL, prepared as described above). Control fruit were inoculated with 10 µL of a 0.05% Tween 20 solution in sterile distilled water. Four apples were inoculated per isolate and incubated for 10 days in plastic containers under high humidity at 20 ± 2 °C. The decay incidence (infected wounds, %) and severity (lesion diameters, mm) were assessed by the end of incubation. Reisolation of the pathogen was performed as previously described to verify Koch’s postulates.

### 2.5. Assessment of the Putative Ability of Penicillium Isolates to Produce Patulin

The isolates were tested for the presence of the 6-methylsalycilic acid synthase (*msas*) gene, involved in the biosynthetic pathway of patulin, using primers Pe11F and Pe12R [29]. PCR mixture (25 μL) contained 50 ng of template DNA, 1X SuperHot MasterMix (Bioron, Römerberg, Germany), and 0.2 μM of each primer. The conditions for the amplification were as follows: initial denaturation at 94 °C for 5 min, 35 cycles at 94 °C for 45 s, 56 °C for 45 s, and 72 °C for 60 s, followed with final elongation for 7 min at 72 °C. PCR products were visualized in 1.5% agarose gel under UV light and the presence/absence of the amplicon corresponding to a 288 bp portion of the *msas* gene was recorded.

### 2.6. Patulin Production of Penicillium Isolates

Patulin production by *Penicillium* isolates was determined using apple fruit from a pathogenicity assay. Apple tissue cylinders (10 mm width × 20 mm depth) were withdrawn from the lesions of each replicate using a cork borer. Tissue samples were pooled together and then homogenized and treated according to Sanzani et al. [30]. Briefly, a test portion (10 g) of each homogenized sample was treated with 10 drops of a pectinase enzyme solution (≥3800 units/mL, Merck) in presence of 10 mL of water. After overnight enzymatic digestion at room temperature, samples were centrifuged at 3900× *g* for 10 min, filtered through a filter paper and a 0.45 μm PTFE syringe filter and analyzed by HPLC.

HPLC analysis was conducted using a mobile phase that consisted of an acetic acid water solution (0.35 M)/acetonitrile (92/8 *v*/*v*). The quantifications were performed by measuring the area of the chromatographic peaks, as previously described. Patulin was quantified by injecting 50 µL of the filtrate extract into a liquid chromatographer (Shimadzu corporation, Kyoto, Japan) equipped with a binary gradient pump capable of delivering a 1 mL/min constant flow rate (Nexera X2 series gradient pump LC30AD), a vacuum membrane degasser (DGU-20A3R 3 channel), an autosampler injection system with a 50 µL loop (SIL-30A/C), a column oven set at 30 °C, diode array detector (DAD, SPD-M30A) set at 276 nm, a chromatography data system for Windows 7 (data analysis), and a Luna (Phenomenex, Torrance, CA, USA) C18 TMS endcapping column (25 cm l., 4.6 mm i.d., 5.0 µm particle size) (Phenomenex, Torrance, CA, USA). Five calibrant solutions were prepared in the range 1–20 μg/mL by diluting the stock standard solution in acidified distilled water. Results were reported as μg of patulin per g of fresh apple weight.

### 2.7. Statistical Analysis

TIBCO Statistica software version 13.3.0 (TIBCO Software Inc, Palo Alto, CA, USA) was used for statistical analysis. Normality of data distribution was checked using the Shapiro–Wilk test, and homogeneity of variances was assessed using the Levene test. Although these tests showed that some of the data were not normally distributed and that in some cases variances were not homogenous, parametric tests were used due to large sample sizes [31,32]. One-way ANOVA was used to determine if there was a difference in colony size between isolates, species, or between culture media and the difference in lesion size between isolates. Post hoc Tukey HSD test was used to assess the differences that emerged during multiple group testing. Spearman correlation was used to assess the putative correlation between pathogenicity (lesion size) and patulin production.

### 2.8. Principal Component Analysis

To elucidate relationships among the characteristics of *Penicillium* isolates, a Principal Component Analysis (PCA) using PAST 4.17 software [33] was conducted. Morphological characteristics (colony diameter on all tested media; reverse color on PDA, MEA, and CYA, and exudate production on CYA) together with pathogenicity and patulin production were assessed for all 70 isolates. Prior to analysis, data were z-standardized using the following formula: z = (x − μ)/σ.

## 3. Results

### 3.1. Sample Collection and Fungal Isolation

**Figure 1:**
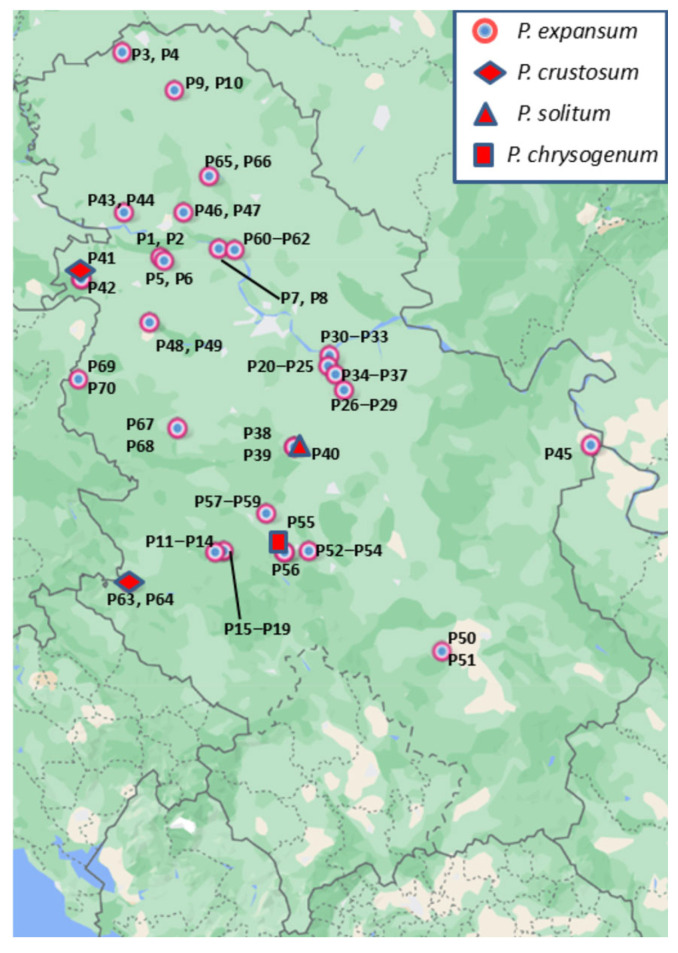
Map of Serbia with locations where *Penicillium* spp. isolates were obtained.

Typical symptoms of blue mold were recorded on naturally infected apple fruit collected from 26 locations in Serbia (Figure 1). Primarily, soft, watery circular lesions, light tan to brown, readily detaching from the healthy part of the fruit, were observed. Commonly, blue-green spore masses covered the surface of decayed tissue. Seventy isolates were obtained, which micromorphologically formed single-celled conidia, ellipsoidal to globose in shape, on brush-like branched conidiophores. These characteristics matched those of *Penicillium* spp.

### 3.2. Morphological Analysis

Micromorphology on MEA was observed. Particularly, 65 isolates formed terverticillate conidiophores with stipes that were cylindrical, usually smooth-walled, and sometimes rough-walled at the basal part. Conidia were ellipsoidal, smooth-walled, and blue-green to grey-green. Metulae and rami were cylindrical, phialides from cylindrical to ampulliform. These characteristics matched those of *P. expansum.* Three isolates (P41, P63, and P64) formed terverticillate conidiophores with rough-walled stipes. The conidia were smooth-walled, globose to subglobose, and blue green. The metulae, rami, and phialides were cylindrical. Those characteristics matched those of *P. crustosum.* An isolate (P40) formed smooth to slightly rough-walled blue-green conidia on terverticillate conidiophores with cylindrical phialides, metulae, and rami, matching features of *P. solitum*. An isolate (P55) formed smooth-walled blue green conidia, on ter- and quarterverticillate conidiophores with smooth-walled stipe. The phialides were cylindrical with short, broad collula, and the metulae and rami were cylindrical. Those characteristics matched those of *P. chrysogenum.*

**Figure 2:**
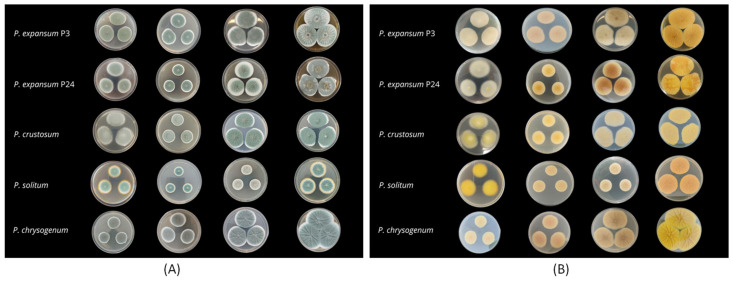
Colony characteristics, front (**A**) and reverse (**B**), of representative isolates *of Penicillium expansum* (P3, P24), *Penicillium crustosum* (P64), *Penicillium solitum* (P40), and *Penicillium chrysogenum* (P55) on different growth media (left to right: PDA, MEA, CYA, and YES) at 7 days post-inoculation at 25 ± 1 °C.

Culture characteristics of the isolates were evaluated on different growth media. *Penicillium expansum* isolates formed white mycelia with blue green conidia on all media. On PDA, the colonies were fasciculate, with entire margins. The colony reverse was creamy or yellow, and no exudate was observed. On MEA, the colonies were fasciculate, sometimes with concentric zones and radially sulcate, with clear exudate droplets on the edges. The colony reverse was mostly cream, yellow in some cases (P8, P13, P15, P17, P24, P27, and P32). On CYA, *P. expansum* formed dense, concentrically fasciculate colonies, sometimes velutinous. In some isolates, exudate was absent, while others produced scarce or abundant droplets. Colony reverse varied from cream, yellow, salmon pink/orange pink to brown. The colony reverse of isolate P30 was orange with brown center. On YES, the colonies were fasciculate or velutinous, sometimes with concentric zones, radially sulcate, with exudate. The color of the colony front varied from blue-green to grey and the reverse from pale to intense yellow, sometimes with an orange center (Figure 2).

Isolates of *P. crustosum* formed white mycelia with blue, dull green conidia on all media. Colonies on PDA and MEA were velutinous with white margins, and no exudate was present, and the reverse was yellow. On CYA, the colonies were velutinous, radially sulcate, with scarce exudate droplets and cream reverse. On YES, the colonies were dense, velutinous, radially sulcate, with no exudate and an intense yellow reverse (Figure 2).

*Penicillium chrysogenum* formed white mycelia with blue green conidia on all media. The colonies on PDA and MEA were velvety with white margins. The reverse color was yellow on PDA and salmon on MEA. On CYA, the colonies were velvety, radially sulcate with brown reverse, without exudate. On YES, colonies were velvety, radially sulcate, with citrine yellow reverse and no exudates (Figure 2).

*Penicillium solitum* formed white mycelia with blue green conidia on all media. On PDA, the colonies were velutinous, with orange reverse and without exudate. On MEA, the colonies were floccose and radially sulcate, without exudate. Colony reverse was orange with yellow margin. On CYA, the colonies were floccose and radially sulcate, with abundant clear exudate droplets. Colony reverse was cream with a bright orange center. On YES, the colonies were floccose and radially sulcate with bright blue conidia. The exudate was absent. Colony reverse was intense yellow (Figure 2).

The colony growth rates of isolates on different media is shown in Supplementary Table S2. The most intensive growth of all isolates was observed on YES medium. *Penicillium solitum* was the slowest growing species on all tested media (*p* < 0.01). *Penicillium chrysogenum* with a mean colony diameter of 26.2 mm on PDA significantly differed from the *P. solitum* isolate and all *P. crustosum* and *P. expansum* isolates (*p* < 0.01).

A vast variability in colony growth was observed among *P. expansum* isolates on all media (*p* < 0.01). On PDA, the *P. expansum* colony diameter was 48.4 mm on average, however, the slowest growing isolate, P62, developed colonies of 32.7 mm on average, while the fastest growing was isolate P20, with 55.7 mm. Similarly, the average colony diameter of *P. expansum* on MEA was 27.39 mm, with slower growing isolates P15 and P62 (23 mm) and the fastest growing isolate P30 (35.3 mm). On CYA, the mean colony size for *P. expansum* was 44.3 mm, while P23 and P24 formed the smallest colonies with 35 mm in diameter, while the biggest colony size was recorded for P2 (51.7 mm). On YES, the average colony diameter was 54.4 mm, but the smallest colonies were observed for P33 (44.5 mm) and the biggest for P50 (62.7 mm). The average colony size of *P. crustosum* isolates were as follows: 36.6 mm on PDA, 26.3 mm on MEA, 41.1 mm on CYA, and 47.8 on YES, with significant variability among isolates detected on MEA (*p* < 0.01).

### 3.3. Molecular Identification and Phylogeny

Molecular identification was performed using the two molecular markers *BenA* and *CaM*. The phylogenetic analysis using concatenated sequences clustered readily and consistently the isolates from the present study with reference strains: one isolate (P55) clustered with *P. chrysogenum*, one (P40) with *P. solitum*, three (P41, P63, and P64) with *P. crustosum*, and 65 (P1–P39, P42–P54, P56–P62, P65–P70) with *P. expansum*. Molecular identification confirmed *P. expansum* as the major cause of apple blue mold in this study (92.9%), followed by *P. crustosum* (4.3%), *P. solitum* (1.4%), and *P. chrysogenum* (1.4%).

Based on sequence alignment of *P. expansum* strains and BLAST analysis, Single-Nucleotide Polymorphisms (SNPs) were found in *BenA* sequences at position 86, 109, 208, 209, and 237. As such strains could be divided into five different groups with 1, 2 or 3 SNPs (Table 1). The first *BenA* variant (58 isolates) had 100% sequence similarity with several *P. expansum* sequences available in the GenBank (i.e., MZ364047 of a *P. expansum* strain isolated from apple fruit in Serbia and JX091539 from the Fynbos biome in South Africa). The second (one isolate, P67) had a transition (A/G) at position 86. The third (two isolates, P23 and P24) had two transitions (A/G and C/T) at position 109 and 237, respectively, and one transversion (T/G) at position 209. The fourth variant (one isolate, P19) had a transition (A/G) and a transversion (T/G) at position 208 and 209, respectively. The fifth *BenA* variant (three isolates, P15, P17, and P62) was identical with several available sequences (i.e., MW162405 from pear fruit in Serbia and MK451084 from plum in South Africa), with a transition (A/G) and a transversion (T/G) at position 209 and 237, respectively.

Two variants in *CaM* sequences were present involving 3 SNPs. Three transitions of T/C (2) and A/G (1) were present at position 68, 143, and 345, respectively. The first *CaM* variant (62 isolates) had sequences identical with numerous Genebank sequences of relevant strains (i.e., MZ364100 from apple fruit in Serbia and MG714821 from sugar beet in the USA). No variants were detected in *BenA* and *CaM* sequences of *P. crustosum* isolates (P41, P63, and P64). Sequences obtained in this study were identical to several sequences of *Penicillium* isolates from Serbia (i.e., from apple fruit: *BenA*: MZ364068 and *CaM*: MZ389061 and from pear fruit: *BenA*: MW162402 and *CaM*: MW115930.1). *Penicillium solitum* (P40) *BenA* and *CaM* sequences were identical with MN149926 and MN149945 of unknown origin, while those of *P. chrysogenum* (P55) were identical with sequences of the strain CBS 132,217 from an indoor environment in Canada (JX996871 and JX996211).

**Figure 3:**
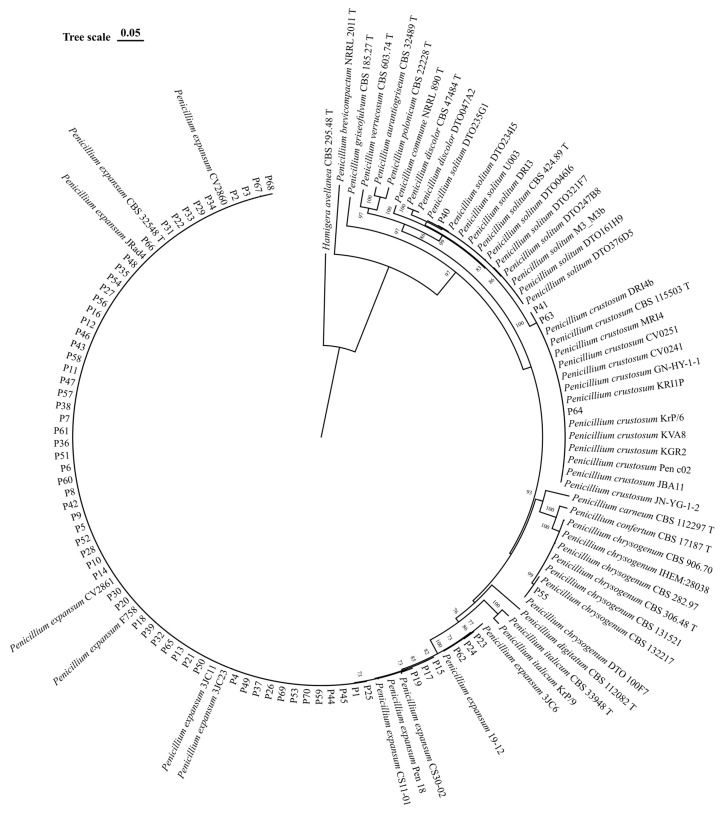
Phylogenetic tree based on the concatenated *BenA* and *CaM* sequences of reference strains of *Penicillium* spp. and the isolates from this study. Numbers on nodes represent the maximum likelihood bootstrap percentages (values higher than 70% are shown at relevant branches). Branch lengths are proportional to the number of nucleotide substitutions and measured using the bar scale (0.050). Type strains are indicated with a T.

The combined dataset of the concatenated two single locus alignments (*BenA* and *CaM*) contained 818 characters. In the phylogenetic tree constructed using the ML method with the Tamura–Nei model, isolates of *P. expansum*, *P. crustosum*, *P. solitum*, and *P. chrysogenum* from this study clustered together with the other reference sequences of corresponding species with bootstrap values > 90 (Figure 3).

### 3.4. Pathogenicity Assay

**Figure 4:**
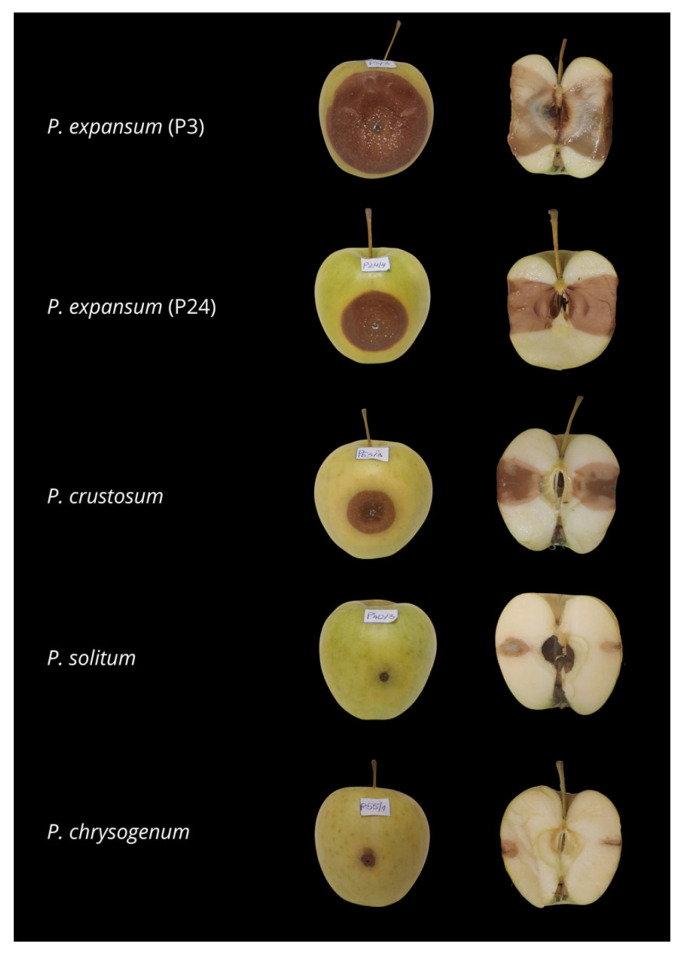
Pathogenicity of *Penicillium expansum* (P3 and P24), *Penicillium crustosum* (P64), *Penicillium solitum* (P40), and *Penicillium chrysogenum* (P55) on artificially inoculated apple fruit, after 10 days of incubation at 20 ± 2 °C.

All isolates were pathogenic and caused rot symptoms on inoculated apple fruit at 10 days post-inoculation (Figure 4), while the control fruit did not show any symptoms. The symptoms on artificially inoculated fruit were similar to those observed in original hosts and appeared as soft, brown, sunken, watery lesions. Blue-green sporulation was noticed in fruit infected with all species except *P. chrysogenum*. On cross sections of inoculated fruit, it was observed that *P. crustosum* induced a slightly darker color of the infected inner tissue. Reisolated fungi showed identical morphological characteristics as original isolates used for inoculation, thus confirming Koch’s postulates.

**Figure 5:**
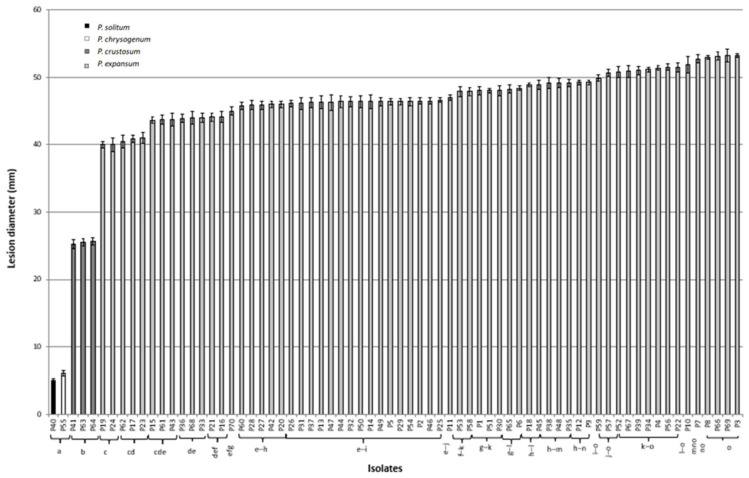
Pathogenicity of *Penicillium* spp. in terms of lesion diameter (mm) on artificially inoculated apple fruit, after 10 days of incubation at 20 ± 2 °C. Data are the mean of four replicates ± standard error of the mean (SEM). Bars with different letters are statistically significant (*p* ≤ 0.05) according to Tukey HSD test.

Based on the lesion size, statistically significant differences (*p* ≤ 0.05) were observed in pathogenicity among isolates. The smallest lesions were caused by *P. solitum* (P40) and *P. chrysogenum* (P55): 5 and 6.1 mm on average, respectively. Three isolates of *P. crustosum* (P41, P63, and P64) with an average lesion size of 25 mm significantly differed from other species. The mean lesion size induced by *P. expansum* isolates was 47 mm. However, significant variability in pathogenicity was observed among *P. expansum* isolates. Isolates P19 and P24 induced smaller lesions (40 mm), while the most severe decay was caused by isolates P3 and P69 (53.2 mm) (Figure 5).

### 3.5. Characterization of the Ability of Penicillium Isolates to Produce Patulin

The presence of a 288 bp amplicon, corresponding to a portion of the *msas* gene involved in the biosynthetic pathway of patulin, was detected in all *P. expansum* isolates, whereas the amplicon was not detected in isolates of *P. crustosum*, *P. solitum*, and *P. chrysogenum* (Supplementary Table S3).

The patulin concentrations were determined by HPLC analysis (Supplementary Table S3). Patulin was detected in all apple samples infected by *P. expansum* isolates, with concentrations ranging from 0.17 (P5) to 56.44 (P24) µg/g. The average patulin concentration in those samples was 10.3 µg/g. Patulin was not detected in samples infected by *P. crustosum*, *P. solitum*, and *P. chrysogenum*.

The spearman correlation analysis between the patulin concentration in apple samples infected by *P. expansum* strains and the pathogenicity of the same strains showed no correlation between the two parameters.

### 3.6. PCA Analysis

**Figure 6:**
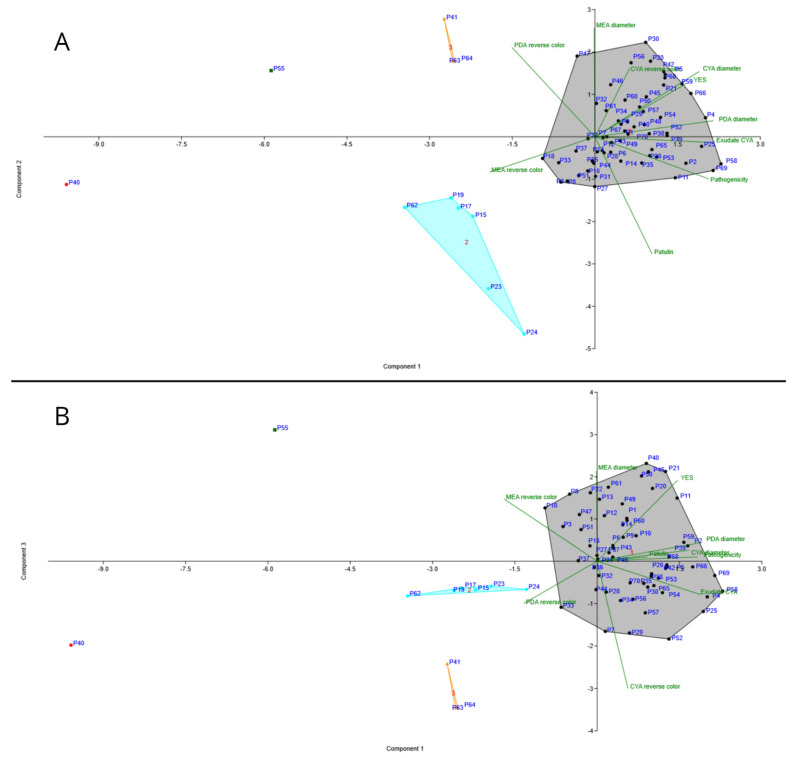
Projection of *Penicillium* isolates based on the following: (**A**) first and second principal component and (**B**) first and third principal component according to Principal Component Analysis (PCA) of isolate characteristics (morphology, colony growth, pathogenicity, and patulin production). *Penicillium expansum* isolate groups are 1 (grey) and 2 (blue), while *P. crustosum* isolate group is orange, P40 is *P. solitum* isolate, and P55 is *P. chrysogenum* isolate.

The PCA analysis separated all 70 isolates based on morphological data and data on pathogenicity and patulin production in three groups (Figure 6A). The total variability of 84.6 was explained by the first and the second component. Principal component 1 (PC1) accounts for 57.3% of the variance and was positively associated with growth characteristics (colony diameters on PDA and CYA) and pathogenicity. It distinctively separates slower growing, less pathogenic isolates (left) from those with larger colonies and higher virulence (right). PC2 accounts for 27.5% of variance, and it is strongly positively correlated with colony diameters on MEA and reverse color on PDA (yellow coloration has greater value than cream) and negatively correlated with patulin production. It separates isolates that grow faster on MEA and have darker reverse color on PDA (up). The third component PC3 (Figure 6B) accounts for 15.4% of variance, and it is strongly positively correlated with exudate production and reverse color on CYA. Isolates of *P. crustosum* (P41, P63, and P64) formed a separate cluster indicating that they share characteristics contributing mainly to PC2. The distinct position of isolates P40 (*P. solitum*) and P55 (*P. chrysogenum*) indicate their unique characteristics that set them apart from *P. expansum* and *P. crustosum* isolates. Separation of *P. expansum* isolates into two distinctive groups was in accordance with polymorphysm in *BenA* and *CaM* sequences, with the exception of P67 (single nucleotide variant in *BenA* sequence).

## 4. Discussion

This research presents the data regarding causal agents of apple blue mold in Serbia. Besides the identification of *Penicillium* species associated with apple blue mold, a pathogenicity assay was performed to gain insights about the virulence of the collected isolates and to confirm Koch’s postulates. Moreover, macromorphology, growth on different media and patulin production potential of all isolates, was tested. The causal agents of apple blue mold were identified as *P. expansum*, *P. crustosum*, *P. solitum*, and *P. chrysogenum*, which is consistent with previous studies [6,7,8,9,10,11,34]. However, this is the first finding of *P. solitum* and *P. chrysogenum* on apple fruit in Serbia, while *P. expansum* and *P. crustosum* have been elaborately studied [16,17]. In Serbia, *P. solitum* has been recently described as a pathogen on quince [17].

The most common causal agent of apple blue mold was *P. expansum*, followed by *P. crustosum*, and these findings correlate with existing studies [17]. Sanderson and Spotts [9] found a variety of *Penicillium* species associated with blue mold on apples and pears in Oregon and Washington State (*P. auarantiogriseum*, *P. commune*, *P. solitum*, *P. verrucosum*, and *P. expansum)*. In British Columbia, *P. brevicompactum*, *P. crustosum*, and *P. expansum* were reported as causal agents of apple blue mold [10], while in Uruguay, *P. expansum* and *P. solitum* were isolated from decayed apple fruit [11]. In China, *P. expansum*, *P. crustosum*, and *P. polonicum* were reported [12], while in Pakistan, only *P. expansum* has been recorded [35]. In Lebanon, Habib et al. [34] reported *P. expansum* as major causal agent of apple blue mold, followed by *P. solitum*. Our research is in accordance with these data regarding *Penicillium* spp. diversity as cosmopolite pathogens of stored apple fruit, with *P. expansum* being the most common.

In the pathogenicity assay, *P. expansum* isolates were found to be more virulent than *P. crustosum*, *P. solitum*, and *P. chrysogenum*. This is in accordance with findings from previous studies confirming that *P. expansum* is the most virulent species of *Penicillium* on apple fruit [7,9,12,17]. However, significant differences in virulence were observed among *P. expansum* isolates, and pathogenicity was detected as one of major contributors to PC1 in Principal Component Analysis. Although the majority of *P. expansum* isolates showed similar levels of virulence on artificially inoculated apple fruit, several isolates (P15, P17, P19, P23, P24, and P62) were remarkably less virulent. This is in contrast with Morales et al. [36] reporting that variability regarding the ability of *P. expansum* to colonize apple tissue is low.

Furthermore, *Penicillium* spp. isolates were morphologically characterized. The most favorable media for the colony growth of all species was YES, as described by Frisvad and Samson [37]. Colony growth on PDA and CYA, together with pathogenicity, were the major contributors of PC1 in PCA, while major contributors to PC2 were colony diameter on MEA and reverse color of PDA. Based on these characteristics, two distinctive groups of *P. expansum* isolates were observed: slower growing, less pathogenic ones (P15, P17, P19, P23, P24, and P62) and the faster growing, more virulent ones (the rest). This grouping was in accordance with polymorphisms in *BenA* and *CaM* sequences, with the exception of P67 (single nucleotide difference in *BenA* sequence). A similar type of grouping was reported by Žebeljan et al. [17] while correlation in the variability of morphological and genetic characteristics has also been reported [38,39].

Finally, the patulin production potential of *Penicillium* isolates was assessed by molecular detection of the *msas* gene involved in patulin production and HPLC analysis of artificially inoculated apple fruit. A cluster of 15 genes that encode 10 biosynthetic enzymes has been reported to be involved in patulin biosynthesis, namely patL (transcription factor), patK (6-methylsalicylic acid synthase), patG (6-methylsalicylic acid decarboxylase), patH (m-cresol methyl hydroxylase), patI (m-hydroxybenzyl alcohol hydroxylase), patO (isoamyl alcohol oxidase), patJ (unknown), patN (isoepoxydon dehydrogenase), patF (unknown), patD (alcohol dehydrogenase), and patE (glucose-methanol-choline oxidoreductase family protein) [40,41,42,43]. The presence of the *msas* gene was confirmed in all *P. expansum* isolates, while the gene was absent in *P. crustosum*, *P. solitum*, and *P. chrysogenum*. The presence of the gene corresponded to the actual patulin production, thus suggesting the molecular assay as an effective predictive tool. Patulin was detected in all samples of apple tissue inoculated with *P. expansum* in various amounts while it was not detected in samples infected by *P. crustosum*, *P. solitum*, and *P. chrysogenum*. Many species are reported as patulin producers; however, Frisvad [44] questioned the reliability of these findings based on sporadic cases of detection, low amounts of patulin detected, and unavailability of the putative patulin-producing strains to the scientific community for further research. According to Frisvad [44], *P. chrisogenum*, *P. crustosum*, and *P. solitum* are unable to produce patulin. However, *P. solitum* might act as a predisposing agent, enabling the entry of *P. expansum* and resulting in more destructive infections [44]. Patulin is not a prerequisite for *P. expansum* infection, but recent findings suggested it could contribute to virulence depending on the cultivar [41,42,45,46,47,48], although no correlation was observed in the present investigation.

## 5. Conclusions

Our study revealed *P. solitum* and *P. chrysogenum* as causal agents of apple blue mold in Serbia for the first time. However, *P. expansum* was the most frequently isolated and aggressive, followed by *P. crustosum*, which is in accordance to previous findings, while *P. solitum* and *P. chrysogenum* were found sporadically and were less virulent. Furthermore, our study gave insights into morphological and genetic characteristics, as well as the pathogenicity of *Penicillium* spp. associated with apple blue mold. Finally, data on the patulin production potential of *Penicillium* spp. related to apple blue mold are especially significant considering the toxicity of patulin as well as the safety of apple fruit and apple-based products.

## Figures and Tables

**Table 1:** Variant analysis of Single-Nucleotide Polymorphisms (SNPs) of *Penicillium expansum* strains.

Sequence	Position				
*BenA*	86	109	208	209	237
I variant	A	A	G	T	C
II variant	G	A	G	T	C
III variant	A	G	G	G	T
IV variant	A	A	A	G	C
V variant	A	A	G	G	T
*CaM*	68	143	345		
I variant	T	C	A		
II variant	C	T	G		

Gray background represents nucleotide differences amongst variants.

## Data Availability

All relevant data are presented in the manuscript and in the Supplementary Materials. Any further inquiries can be directed to the corresponding author.

## Supplementary Materials

The following supporting information can be downloaded at https://www.mdpi.com/article/10.3390/jof11030175/s1; Table S1: *Penicillium* strains used in this study; Table S2: colony growth; Table S3: patulin production (µg/g) and presence of *msas* gene in *Penicillium* isolates. Refs. [49,50,51,52,53,54,55,56,57,58,59,60,61,62,63,64,65] are cited in the Supplementary Materials.
