# Supplementary material for: Diversity and Patulin Production of Penicillium spp. Associated with Apple Blue Mold in Serbia
Source: J Fungi (Basel). 2025 Feb 21;11(3):175. doi: 10.3390/jof11030175 (PMC11942967; doi:10.3390/jof11030175)
Supplement: Supplementary file 1 [file jof-11-00175-s001.zip › Supplementary Table S1..pdf]

Supplementary Table S1. *Penicillium* spp. strains used in this study for phylogenetic analyses.

Strain	Origin	Gen bank accession number		Reference
		BenA	CaM	
Penicillium expansum				
P1	Serbia, Mala Remeta / Apple, cv. Fuji	PQ787344	PQ787414	This study
P2	Serbia, Mala Remeta / Apple, cv. Fuji	PQ787345	PQ787415	This study
P3	Serbia, Tavankut / Apple, cv. Golden Delicious	PQ787346	PQ787416	This study
P4	Serbia, Tavankut / Apple, cv. Golden Delicious	PQ787347	PQ787417	This study
P5	Serbia, Jazak / Apple cv. Red Delicious	PQ787348	PQ787418	This study
P6	Serbia, Jazak / Apple cv. Gala	PQ787349	PQ787419	This study
P7	Serbia, Krčedin / Apple cv. Golden Delicious	PQ787350	PQ787420	This study
P8	Serbia, Krčedin / Apple cv. Golden Delicious	PQ787351	PQ787421	This study
P9	Serbia, Tornjoš / Apple cv. Golden Delicious	PQ787352	PQ787422	This study
P10	Serbia, Tornjoš / Apple cv. Golden Delicious	PQ787353	PQ787423	This study
P11	Serbia, Arilje / Apple cv. Idared	PQ787354	PQ787424	This study
P12	Serbia, Arilje / Apple cv. Idared	PQ787355	PQ787425	This study
P13	Serbia, Arilje / Apple cv. Golden Delicious	PQ787356	PQ787426	This study
P14	Serbia, Arilje / Apple cv. Golden Delicious	PQ787357	PQ787427	This study
P15	Serbia, Stupčevići / Apple cv. Idared	PQ787358	PQ787428	This study
P16	Serbia, Stupčevići / Apple cv. Idared	PQ787359	PQ787429	This study
P17	Serbia, Stupčevići / Apple cv. Idared	PQ787360	PQ787430	This study
P18	Serbia, Stupčevići / Apple cv. Idared	PQ787361	PQ787431	This study
P19	Serbia, Stupčevići / Apple cv. Idared	PQ787362	PQ787432	This study
P20	Serbia, Vodanj / Apple cv. Granny Smith	PQ787363	PQ787433	This study
P21	Serbia, Vodanj / Apple cv. Granny Smith	PQ787364	PQ787434	This study
P22	Serbia, Vodanj / Apple cv. Granny Smith	PQ787365	PQ787435	This study
P23	Serbia, Vodanj / Apple cv. Red Jonaprince	PQ787366	PQ787436	This study
P24	Serbia, Vodanj / Apple cv. Red Jonaprince	PQ787367	PQ787437	This study

P25	Serbia, Vodanj / Apple cv. Red Jonaprince	PQ787368	PQ787438	This study
P26	Serbia, Dobri Do / Apple cv. Golden Delicious	PQ787369	PQ787439	This study
P27	Serbia, Dobri Do / Apple cv. Golden Delicious	PQ787370	PQ787440	This study
P28	Serbia, Dobri Do / Apple cv. Golden Delicious	PQ787371	PQ787441	This study
P29	Serbia, Dobri Do / Apple cv. Golden Delicious	PQ787372	PQ787442	This study
P30	Serbia, Udovice / Apple cv. Idared	PQ787373	PQ787443	This study
P31	Serbia, Udovice / Apple cv. Idared	PQ787374	PQ787444	This study
P32	Serbia, Udovice / Apple cv. Idared	PQ787375	PQ787445	This study
P33	Serbia, Udovice / Apple cv. Idared	PQ787376	PQ787446	This study
P34	Serbia, Lunjevac / Apple cv. Idared	PQ787377	PQ787447	This study
P35	Serbia, Lunjevac / Apple cv. Idared	PQ787378	PQ787448	This study
P36	Serbia, Lunjevac / Apple cv. Idared	PQ787379	PQ787449	This study
P37	Serbia, Lunjevac / Apple cv. Idared	PQ787380	PQ787450	This study
P38	Serbia, Topola (Vinča) / Apple cv. Golden Delicious	PQ787381	PQ787451	This study
P39	Serbia, Topola (Vinča) / Apple cv. Golden Delicious	PQ787382	PQ787452	This study
P42	Serbia, Morović / Apple cv. Golden Delicious	PQ787383	PQ787453	This study
P43	Serbia, Čelarevo / Apple cv. Braeburn	PQ787384	PQ787454	This study
P44	Serbia, Čelarevo / Apple cv. Granny Smith	PQ787385	PQ787455	This study
P45	Serbia, Negotin / Apple cv. Braeburn	PQ787386	PQ787456	This study
P46	Serbia, Kać / Apple cv. Golden Delicious	PQ787387	PQ787457	This study
P47	Serbia, Kać / Apple cv. Golden Delicious	PQ787388	PQ787458	This study
P48	Serbia, Majur / Apple cv. Granny Smith	PQ787389	PQ787459	This study
P49	Serbia, Majur / Apple cv. Granny Smith	PQ787390	PQ787460	This study
P50	Serbia, Prokuplje / Apple cv. Red Delicious	PQ787391	PQ787461	This study
P51	Serbia, Prokuplje / Apple cv. Golden Delicious	PQ787392	PQ787462	This study
P52	Serbia, Kovanluk / Apple cv. Idared	PQ787393	PQ787463	This study
P53	Serbia, Kovanluk / Apple cv. Idared	PQ787394	PQ787464	This study
P54	Serbia, Kovanluk / Apple cv. Idared	PQ787395	PQ787465	This study
P56	Serbia, Vrdila / Apple cv. Golden Delicious	PQ787396	PQ787466	This study

P57	Serbia, Čačak / Apple cv. Idared	PQ787397	PQ787467	This study
P58	Serbia, Čačak / Apple cv. Idared	PQ787398	PQ787468	This study
P59	Serbia, Čačak / Apple cv. Idared	PQ787399	PQ787469	This study
P60	Serbia, Novi Slankamen / Apple cv. Golden Delicious	PQ787400	PQ787470	This study
P61	Serbia, Novi Slankamen / Apple cv. Golden Delicious	PQ787401	PQ787471	This study
P62	Serbia, Novi Slankamen / Apple cv. Granny Smith	PQ787402	PQ787472	This study
P65	Serbia, Čurug / Apple cv. Jonagold	PQ787404	PQ787473	This study
P66	Serbia, Čurug / Apple cv. Golden Delicious	PQ787405	PQ787474	This study
P67	Serbia, Valjevo / Apple cv. Golden Delicious	PQ787406	PQ787475	This study
P68	Serbia, Valjevo / Apple cv. Golden Delicious	PQ787407	PQ787476	This study
P69	Serbia, Loznica / Apple cv. Granny Smith	PQ787408	PQ787477	This study
P70	Serbia, Loznica / Apple cv. Granny Smith	PQ787404	PQ787478	This study
CBS 325.48^{T}	USA / Apple fruit	AY674400.1	DQ911134	[49]
JRad4	Serbia, Radmilovac / Apple cv. Gloster	MZ364047	MZ364097	[17]
3JC23	Serbia, Čelarevo / Apple cv. Modi	MZ364049	MZ364100	[17]
3JC6	Serbia, Čelarevo / Apple cv. Braeburn	MZ364056	MZ364098	[17]
3JC11	Serbia, Čelarevo / Apple cv. Granny Smith	MZ364048	MZ364099	[17]
19-12	Serbia / Pear fruit	MW162405	MW115931	[50]
CV 2860	South Africa / Fynbos biome	JX091539.1	JX141580	[51]
CV 2861	South Africa / Fynbos biome	JX091540.1	JX141581.1	[51]
Pen 18	Pakistan / Grape	MT387285	MT387295	[52]
F758	USA, Idaho / Sugar beet	MG714864	MG714821	[53]
CS30-02	China, Sichuan / soil	OR051052	OR051231	[54]
CS11-01	China: Chongqing / soil in a cave	OR051048	OR051227	[54]
Penicillium crustosum				
P41	Serbia, Morović / Apple cv. Golden Delicious	PQ787409	PQ787479	This study
P63	Serbia, Priboj / Apple cv. Golden Delicious	PQ787410	PQ787480	This study
P64	Serbia, Priboj / Apple cv. Golden Delicious	PQ787411	PQ787481	This study
CBS 115503^{T}	Scotland, Aberdeen / Lemon	AY674353[1]	DQ911132[2]	[1][55]; [2][49]
JBA11	Serbia, Bavanište / Apple cv. Šifra	MZ364068	MZ389061	[17]

KGR2	Serbia, Grocka / Pear cv. Santa Maria	MZ364063	MZ389062	[17]
KRI1P	Serbia, Ritopek / Pear cv. Williams	MZ364064	MZ389063	[17]
KVA8	Serbia, Valjevo / Pear cv. Poire de Cure	MZ364065	MZ389064	[17]
DRI4b	Serbia, Ritopek / Quince cv. Leskovačka	MZ364066	MZ389065	[17]
MRI4	Serbia, Ritopek / Medlar local cv.	MZ364069	MZ389066	[17]
KrP/6	Serbia / Pear fruit	MW162402	MW115930	[50]
CV0241	South Africa / Fynbos biome	JX091536	JX141576	[51]
CV0251	South Africa / Fynbos biome	JX091530	JX141577	[51]
Pen c02	Pakistan / Grape	MT387257	MT387267	[52]
JN-YG-1-2	Korea / *Tribolium castaneum*	MH424000	MH423988	[56]
GN-HY-1-1	Korea / *Tribolium castaneum*	MH424001	MH423989	[56]
Penicillium solitum				
P40	Serbia, Topola (Vinča) / Apple cv. Golden Delicious	PQ787412	PQ787482	This study
CBS 424.89[T]	Germany/Unknown	AY674354[1]	KU896851[2]	[1][55]; [2][49]
DRI3	Serbia, Ritopek / Quince cv. Leskovačka	MZ364070	MZ364115	[17]
U003	Croatia / Surface of fermented meat	OQ205202	OQ205239	[57]
DTO046I6	Unknown	MN149919	MN149938	[58]
DTO161H9	Unknown	MN149924	MN149943	[58]
DTO234I5	Unknown	MN149926	MN149945	[58]
DTO_235G1	Australia / Indoor house dust	KJ775163[1]	MN149946[2]	[1][20]; [2][58]
DTO247B8	Unknown	MN149927	MN149947	[58]
DTO321F7	Unknown	MN149928	MN149948	[58]
DTO376D5	Unknown	MN149930	MN149950	[58]
M3_M3b	Italy / Cheese rind	LR745785	LR745841	[59]
Penicillium chrysogenum				
P55	Serbia, Vrdila / Apple cv. Jonagold	PQ787413	PQ787483	This study
CBS 306.48	USA, Connecticut	JF909955	JX996273	[49]
CBS 906.70	The Netherlands / Culture contaminant	JX996934	JX996284	[60]
CBS 282.97	South Africa / Barley	JX996925	JX996271	[60]
CBS 132217	Canada / Indoor environment	JX996871	JX996211	[60]
CBS 131521	Netherlands, Utrecht / Ceiling in archive	JF909946	JF909964	[61]
DTO 100-F7	Unknown	JX996860	JX996199	[61]

IHEM:28038	Belgium, Puurs / Bat (*Plecotus auritus* fur	OU641458	OU641459	[62]
Penicillium italicum				
CBS 339.48[T]	USA / Citrus fruit	[1]AY674398	[2]DQ911135	[1][40]; [2][48]
KrP/9	Serbia / Pear fruit	MW162410	MW115932	[35]
Penicillium discolor				
CBS 474.84[T]	Israel / *Raphanus sativus*	[1]AY674348	[2]KU896834	[1][55]; [2][49]
DTO047A2	Unknown	MN149922	MN149941	[58]
Penicillium confertum				
CBS 171.87[T]	USA, Arizona / Cheek pouch	[1]AY674373	[2]JX996963	[1][55]; [2][60]
Penicillium aurantiogriseum				
CBS:324.89[T]	Belgium / Unknown	AY674296	[2]KU896822	[1][55]; [2][64]
Penicillium brevicompactum				
NRRL 2011[T]	Unknown	AY674437	AY484817	[20]
Penicillium carneum				
CBS 112297[T]	Unknown	AY674437	HQ442322	[20]
Penicillium commune				
CBS 311.48[T]	USA / Cheese	[1]MN969377	KU896829	[1][49]; [2][64]
Penicillium digitatum				
CBS 112082[T]	Italy / Lemon	[1]KJ834447	[2]AY484817	[1][20]; [2][65]
Penicillium griseofulvum				
CBS 185.27[T]	Belgium / Soil	JF909942	KT900574	[64]
Penicillium polonicum				
CBS 222.28[T]	Poland / Soil	[1]AY674305	[2]KU896848	[1][55]; [2][64]
Penicillium verrucosum				
CBS 603.74[T]	Poland / Soil	[1]MN969405	[2]DQ911138	[1][49]; [2][63]
Hamigera avellanea				
NRRL 1938[T]	Unknown	EU021664	EU021682	[49]

[T]Type strain.
