# Supplementary material for: Diversity and Patulin Production of Penicillium spp. Associated with Apple Blue Mold in Serbia
Source: J Fungi (Basel). 2025 Feb 21;11(3):175. doi: 10.3390/jof11030175 (PMC11942967; doi:10.3390/jof11030175)
Supplement: Supplementary file 1 [file jof-11-00175-s001.zip › Supplementary Table S2.pdf]

Supplementary Table S2. The colony diameter (mm ± standard error of the mean (SEM)) of *Penicillium* isolates on different media after 7 days of incubation in the dark at 25 ± 1 °C. Numbers with different letters represent a statistically significant difference in colony diameter according to Tukey HSD test ($p < 0.01$).

Isolate	Species	PCA Cluster	PDA			MEA			CYA			YES		
**P1**	*Penicillium expansum*	1	50.7	±1.6	pqr	28.3	±1.0	g-l	45.5	±0.4	jk	55.7	±0.7	ij
**P2**	*Penicillium expansum*	1	50.2	±0.6	pq	26.8	±0.6	c-j	51.7	±0.6	op	52.0	±0.6	g
**P3**	*Penicillium expansum*	1	52.0	±0.4	rst	25.7	±0.2	b-g	39.3	±0.3	c	51.2	±0.4	efg
**P4**	*Penicillium expansum*	1	51.5	±0.7	qrs	25.2	±0.3	b-g	51.5	±0.7	op	54.7	±0.4	ij
**P5**	*Penicillium expansum*	1	48.2	±0.5	mn	31.2	±0.4	m-r	49.3	±0.3	no	53.5	±1.6	hi
**P6**	*Penicillium expansum*	1	47.3	±0.5	lm	27.0	±0.3	d-k	48.0	±0.7	mn	49.5	±0.6	def
**P7**	*Penicillium expansum*	1	48.2	±0.3	mn	26.3	±0.2	c-i	41.5	±0.4	de	47.5	±0.4	c
**P8**	*Penicillium expansum*	1	44.0	±0.6	ijk	27.5	±0.2	e-l	38.7	±0.5	c	59.5	±0.4	mno
**P9**	*Penicillium expansum*	1	52.2	±0.6	rst	26.0	±0.4	b-i	47.8	±0.3	lmn	57.8	±0.6	j-m
**P10**	*Penicillium expansum*	1	53.7	±0.3	uvw	25.2	±0.3	b-g	47.2	±0.3	lm	59.8	±0.7	m-p
**P11**	*Penicillium expansum*	1	52.8	±0.5	tuv	28.5	±0.8	h-m	45.8	±0.6	jkl	60.8	±1.6	pqr
**P12**	*Penicillium expansum*	1	52.0	±0.4	rst	27.5	±0.4	e-l	46.2	±0.3	kl	54.0	±0.7	i
**P13**	*Penicillium expansum*	1	52.0	±0.3	rst	26.8	±0.5	c-j	46.3	±0.5	klm	56.7	±0.4	ij
**P14**	*Penicillium expansum*	1	51.5	±0.4	qrs	28.7	±0.4	i-n	44.2	±0.3	hi	52.7	±0.4	gh
**P16**	*Penicillium expansum*	1	49.7	±0.2	opq	26.0	±0.5	b-i	41.2	±0.3	d	52.3	±1.1	g
**P18**	*Penicillium expansum*	1	48.3	±0.5	mno	26.0	±0.4	b-i	46.7	±0.2	lm	52.5	±0.7	gh
**P20**	*Penicillium expansum*	1	55.7	±0.5	y	35.0	±0.4	st	45.7	±0.3	jk	59.0	±0.6	mn
**P21**	*Penicillium expansum*	1	54.2	±0.6	vwx	32.0	±0.4	p-s	48.8	±0.3	mno	62.5	±1.1	s
**P22**	*Penicillium expansum*	1	54.2	±0.6	vwx	31.3	±5.0	n-r	45.2	±0.3	ij	49.2	±0.7	de
**P25**	*Penicillium expansum*	1	55.0	±0.6	wxy	23.8	±0.3	bc	50.5	±0.2	op	52.0	±0.7	g
**P26**	*Penicillium expansum*	1	46.2	±0.3	j-l	27.8	±0.3	f-l	47.0	±0.4	lm	50.3	±0.4	ef
**P27**	*Penicillium expansum*	1	45.3	±0.2	jk	26.8	±0.4	c-j	42.7	±0.4	fg	51.3	±0.3	efg

**P28**	*Penicillium expansum*	1	42.3	±0.4	hij	27.3	±0.3	d-l	44.3	±0.5	hi	46.8	±0.5	bc
**P29**	*Penicillium expansum*	1	51.5	±0.6	qrs	24.0	±0.4	bcd	44.2	±0.5	hi	51.2	±0.4	efg
**P30**	*Penicillium expansum*	1	49.3	±0.5	opq	35.3	±0.2	t	47.0	±0.6	lm	50.7	±0.6	ef
**P31**	*Penicillium expansum*	1	48.2	±0.7	mn	24.2	±0.3	b-e	43.8	±0.3	ghi	51.5	±0.4	fg
**P32**	*Penicillium expansum*	1	48.7	±0.3	op	28.5	±0.4	h-m	45.8	±0.4	jkl	53.0	±0.6	h
**P33**	*Penicillium expansum*	1	42.0	±0.4	hij	24.5	±0.3	b-g	43.7	±0.3	gh	44.5	±0.4	b
**P34**	*Penicillium expansum*	1	48.2	±0.5	mn	26.2	±0.7	c-i	44.8	±0.3	hij	49.8	±0.4	def
**P35**	*Penicillium expansum*	1	48.3	±0.4	mno	25.7	±0.3	b-g	51.5	±0.4	op	50.8	±0.5	efg
**P36**	*Penicillium expansum*	1	52.7	±0.7	stu	25.5	±0.3	b-g	45.5	±0.2	jk	49.2	±0.5	de
**P37**	*Penicillium expansum*	1	47.7	±0.6	lmn	27.3	±0.8	d-l	45.0	±0.4	ij	48.7	±0.4	de
**P38**	*Penicillium expansum*	1	48.5	±0.2	nop	27.3	±0.5	d-l	46.5	±0.2	lm	50.5	±0.4	ef
**P39**	*Penicillium expansum*	1	47.3	±0.5	lm	28.0	±0.4	f-l	45.7	±0.3	jk	55.7	±0.3	ij
**P42**	*Penicillium expansum*	1	50.5	±0.2	pqr	29.5	±0.4	k-m	46.0	±0.4	kl	61.0	±0.5	qr
**P43**	*Penicillium expansum*	1	51.2	±0.7	p-s	28.7	±0.7	i-n	42.7	±0.4	fg	53.5	±0.4	hi
**P44**	*Penicillium expansum*	1	51.8	±0.6	rst	25.8	±0.3	b-h	42.7	±0.3	fg	49.7	±0.4	def
**P45**	*Penicillium expansum*	1	53.8	±0.3	vwx	33.0	±0.6	rst	43.7	±0.3	gh	61.2	±0.5	qr
**P46**	*Penicillium expansum*	1	50.2	±0.7	pq	28.8	±0.5	j-n	48.7	±0.3	mno	54.0	±0.4	i
**P47**	*Penicillium expansum*	1	50.0	±0.8	pq	31.0	±0.5	m-r	45.7	±0.3	jk	60.7	±0.7	o-r
**P48**	*Penicillium expansum*	1	51.5	±0.6	qrs	34.8	±0.5	st	41.7	±0.3	ef	60.5	±0.8	opq
**P49**	*Penicillium expansum*	1	52.2	±0.5	rst	28.8	±0.6	j-n	40.3	±0.3	cd	60.0	±0.8	nop
**P50**	*Penicillium expansum*	1	46.7	±0.7	klm	32.0	±0.6	p-s	46.3	±0.9	klm	62.7	±0.7	s
**P51**	*Penicillium expansum*	1	46.3	±0.3	j-m	24.0	±0.6	bc	42.8	±0.5	fg	58.3	±0.4	klm
**P52**	*Penicillium expansum*	1	49.8	±0.7	pq	23.8	±0.5	bc	43.5	±0.6	gh	57.5	±0.5	jkl
**P53**	*Penicillium expansum*	1	48.7	±0.3	op	24.2	±0.4	b-e	43.7	±0.3	gh	56.8	±0.3	ijk
**P54**	*Penicillium expansum*	1	50.7	±0.3	pqr	25.7	±0.3	b-g	45.7	±0.3	jk	60.5	±0.3	opq
**P56**	*Penicillium expansum*	1	52.3	±0.5	stu	28.3	±0.3	g-l	45.0	±0.3	ij	57.3	±0.3	i-l
**P57**	*Penicillium expansum*	1	50.3	±0.4	pqr	25.5	±0.4	b-g	45.2	±0.3	ij	58.7	±0.5	lmn
**P58**	*Penicillium expansum*	1	52.5	±0.8	stu	24.3	±0.3	b-f	49.2	±0.6	no	58.0	±0.6	klm
**P59**	*Penicillium expansum*	1	55.2	±0.3	wxy	29.0	±0.7	k-n	45.8	±0.4	jkl	61.7	±0.8	rs
**P60**	*Penicillium expansum*	1	51.3	±0.8	p-s	30.7	±0.3	l-q	41.0	±0.4	d	59.2	±0.3	mn

**P61**	*Penicillium expansum*	1	48.2	±0.5	mn	31.7	±0.5	o-r	42.0	±0.4	f	60.3	±0.6	nop
**P65**	*Penicillium expansum*	1	41.8	±0.3	hi	26.8	±0.5	c-j	43.2	±0.3	g	58.0	±0.6	klm
**P66**	*Penicillium expansum*	1	50.0	±0.6	pq	30.3	±0.5	l-p	47.5	±0.4	lmn	59.0	±0.6	mn
**P67**	*Penicillium expansum*	1	44.7	±0.4	ijk	27.0	±0.3	d-k	42.5	±0.4	fg	56.7	±0.7	ij
**P68**	*Penicillium expansum*	1	51.3	±0.4	p-s	30.2	±0.5	k-o	45.8	±0.7	jkl	60.5	±0.6	opq
**P69**	*Penicillium expansum*	1	49.5	±0.8	opq	26.3	±0.4	c-i	44.5	±0.6	hij	60.2	±0.5	nop
**P70**	*Penicillium expansum*	1	45.7	±0.2	jk	25.5	±0.4	b-g	44.3	±0.3	hi	57.5	±0.4	jkl
**P15**	*Penicillium expansum*	2	36.7	±0.5	efg	23.0	±0.4	b	35.5	±0.5	b	47.5	±0.4	c
**P17**	*Penicillium expansum*	2	39.5	±0.7	fgh	23.8	±0.3	bc	36.2	±0.3	b	46.8	±0.5	bc
**P19**	*Penicillium expansum*	2	37.2	±0.5	efg	24.0	±0.4	bc	35.7	±0.6	b	45.3	±0.6	b
**P23**	*Penicillium expansum*	2	34.2	±0.7	cd	24.2	±0.3	b-e	35.0	±0.3	b	45.8	±0.9	bc
**P24**	*Penicillium expansum*	2	40.2	±0.7	gh	23.2	±0.5	b	35.0	±0.3	b	45.2	±0.7	b
**P62**	*Penicillium expansum*	2	32.7	±0.4	c	23.0	±0.4	b	36.7	±0.7	b	45.3	±0.1	b
**P41**	*Penicillium crustosum*	3	36.8	±0.3	efg	31.0	±0.4	l-r	39.7	±0.4	c	47.8	±0.5	c
**P63**	*Penicillium crustosum*	3	36.7	±2.1	efg	24.0	±0.4	bc	41.7	±0.2	ef	47.5	±0.4	c
**P64**	*Penicillium crustosum*	3	36.3	±2.0	cde	23.8	±0.3	bc	42.0	±0.4	f	48.0	±0.4	d
**P40**	*Penicillium solitum*	Distinct position	19.7	±0.5	a	21.7	±0.2	a	23.2	±0.4	a	33.0	±0.3	a
**P55**	*Penicillium chrysogenum*	Distinct position	26.2	±0.6	b	32.8	±0.5	q-t	43.8	±0.4	ghi	59.2	±0.7	mn
