# Supplementary material for: Diversity and Patulin Production of Penicillium spp. Associated with Apple Blue Mold in Serbia
Source: J Fungi (Basel). 2025 Feb 21;11(3):175. doi: 10.3390/jof11030175 (PMC11942967; doi:10.3390/jof11030175)
Supplement: Supplementary file 1 [file jof-11-00175-s001.zip › Supplementary Table S3.pdf]

Supplementary Table S3. Patulin production (μg/g) and presence of *msas* gene in *Penicillium* isolates (+: present; -: absent).

Isolate	Species	PCA Cluster	Patulin (μg/g)	Presence of a portion of *msas* gene
P1	*Penicillium expansum*	1	3.94	+
P2	*Penicillium expansum*	1	17.65	+
P3	*Penicillium expansum*	1	0.67	+
P4	*Penicillium expansum*	1	3.53	+
P5	*Penicillium expansum*	1	0.17	+
P6	*Penicillium expansum*	1	6.72	+
P7	*Penicillium expansum*	1	2.80	+
P8	*Penicillium expansum*	1	9.40	+
P9	*Penicillium expansum*	1	4.24	+
P10	*Penicillium expansum*	1	2.94	+
P11	*Penicillium expansum*	1	29.38	+
P12	*Penicillium expansum*	1	9.96	+
P13	*Penicillium expansum*	1	5.98	+
P14	*Penicillium expansum*	1	14.70	+
P16	*Penicillium expansum*	1	8.10	+
P18	*Penicillium expansum*	1	2.74	+
P20	*Penicillium expansum*	1	2.50	+
P21	*Penicillium expansum*	1	5.94	+
P22	*Penicillium expansum*	1	4.62	+
P25	*Penicillium expansum*	1	11.32	+
P26	*Penicillium expansum*	1	14.98	+
P27	*Penicillium expansum*	1	15.71	+
P28	*Penicillium expansum*	1	5.97	+
P29	*Penicillium expansum*	1	4.13	+
P30	*Penicillium expansum*	1	9.22	+
P31	*Penicillium expansum*	1	6.60	+
P32	*Penicillium expansum*	1	6.55	+
P33	*Penicillium expansum*	1	6.52	+
P34	*Penicillium expansum*	1	2.09	+
P35	*Penicillium expansum*	1	28.69	+
P36	*Penicillium expansum*	1	17.03	+
P37	*Penicillium expansum*	1	15.17	+
P38	*Penicillium expansum*	1	2.97	+
P39	*Penicillium expansum*	1	7.56	+
P42	*Penicillium expansum*	1	7.66	+
P43	*Penicillium expansum*	1	11.60	+

P44	*Penicillium expansum*	1	19.78	+
P45	*Penicillium expansum*	1	4.81	+
P46	*Penicillium expansum*	1	4.78	+
P47	*Penicillium expansum*	1	0.98	+
P48	*Penicillium expansum*	1	18.57	+
P49	*Penicillium expansum*	1	7.62	+
P50	*Penicillium expansum*	1	10.23	+
P51	*Penicillium expansum*	1	9.38	+
P52	*Penicillium expansum*	1	15.26	+
P53	*Penicillium expansum*	1	5.80	+
P54	*Penicillium expansum*	1	9.86	+
P56	*Penicillium expansum*	1	0.55	+
P57	*Penicillium expansum*	1	14.44	+
P58	*Penicillium expansum*	1	22.15	+
P59	*Penicillium expansum*	1	6.05	+
P60	*Penicillium expansum*	1	6.25	+
P61	*Penicillium expansum*	1	5.33	+
P65	*Penicillium expansum*	1	14.03	+
P66	*Penicillium expansum*	1	16.71	+
P67	*Penicillium expansum*	1	2.60	+
P68	*Penicillium expansum*	1	12.61	+
P69	*Penicillium expansum*	1	24.16	+
P70	*Penicillium expansum*	1	12.92	+
P15	*Penicillium expansum*	2	6.22	+
P17	*Penicillium expansum*	2	5.99	+
P19	*Penicillium expansum*	2	1.83	+
P23	*Penicillium expansum*	2	39.07	+
P24	*Penicillium expansum*	2	56.44	+
P62	*Penicillium expansum*	2	6.81	+
P41	*Penicillium crustosum*	3	0.00	-
P63	*Penicillium crustosum*	3	0.00	-
P64	*Penicillium crustosum*	3	0.00	-
P40	*Penicillium solitum*	Distinct position	0.00	-
P55	*Penicillium chrysogenum*	Distinct position	0.00	-
